# Redeveloping Leadership Training for Higher Trainees in the West Midlands

**DOI:** 10.1192/bjo.2022.145

**Published:** 2022-06-20

**Authors:** Devika Patel, Shay-Anne Pantall, Humaira Aziz, Feroz Nainar

**Affiliations:** 1 Coventry and Warwickshire Partnership NHS Trust, Coventry, United Kingdom; 2 Birmingham and Solihull Mental Health NHS Foundation Trust, Birmingham, United Kingdom; 3 Birmingham Community Healthcare NHS Foundation Trust, Birmingham, United Kingdom

## Abstract

**Aims:**

Many of the competencies that trainees in psychiatry are required to achieve can be linked to leadership in the broadest sense, yet specific training is not often systematically provided. The West Midlands Psychiatry Leadership Development Programme aims to support the acquisition of important leadership skills already set out in the curriculum through provision of high-quality specialist leadership content within the existing programme. Here we present the findings of a scoping exercise exploring the views and attitudes towards leadership training held by higher trainees in psychiatry within the West Midlands.

**Methods:**

All psychiatry higher trainees within West Midlands Deanery were invited to complete an anonymous online survey using Survey Monkey in November 2021. This survey incorporated questions about their preferred learning styles, confidence in their leadership skills and barriers to accessing leadership opportunities, generating both quantitative and qualitative data.

**Results:**

Key results included:

- 37 responses were received. All subspeciality training programmes were represented. Almost half of respondents (46%) were ST6 or above and most were in training full time (84%).
- Trainees expressed a preference for experiential learning about leadership (87%) as well as small group teaching (62%) and interactive workshop style content (62%).
- Awareness of leadership opportunities was typically via their peer group (81%) or clinical supervisor (60%). Only 52% of trainees were aware of leadership opportunities within the Deanery.
- Only 54% felt that existing leadership training met their curriculum requirements. Less than half of trainees (46%) felt confident to evidence their leadership experience within their training portfolio.
- One-fifth of trainees (21%) reported experiencing barriers to leadership development. These included: inadequate awareness of opportunities, lack of senior support, time constraints and difficulty matching interests with available opportunities.

**Conclusion:**

Trainees expressed interest in the redevelopment of a regional leadership training programme which would support them to achieve their curriculum competencies and prepare them for life as a consultant psychiatrist. The new multi-faceted regional leadership programme will offer resources in a variety of formats including webinars, podcasts, optional interactive workshops and action learning sets. It is hoped that this flexible programme, linked to the Medical Leadership Competency Framework, will better meet the needs of higher trainees as they pursue their own personal leadership journeys.

